# Utility of 1,3 β-d-Glucan Assay for Guidance in Antifungal Stewardship Programs for Oncologic Patients and Solid Organ Transplant Recipients

**DOI:** 10.3390/jof7010059

**Published:** 2021-01-17

**Authors:** Marina Machado, Esther Chamorro de Vega, María del Carmen Martínez-Jiménez, Carmen Guadalupe Rodríguez-González, Antonio Vena, Raquel Navarro, María Isabel Zamora-Cintas, Caroline Agnelli, María Olmedo, Alicia Galar, Jesús Guinea, Ana Fernández-Cruz, Roberto Alonso, Emilio Bouza, Patricia Muñoz, Maricela Valerio

**Affiliations:** 1 Clinical Microbiology and Infectious Diseases Department, Hospital General Universitario Gregorio Marañón, 28007 Madrid, Spain; mamenmj@telefonica.net (M.d.C.M.-J.); anton.vena@gmail.com (A.V.); rakelnavarro.rodriguez@gmail.com (R.N.); maribel.zamora.cintas@gmail.com (M.I.Z.-C.); agnelli.carol@gmail.com (C.A.); maria.olmedo.samperio@gmail.com (M.O.); aliciagalar@gmail.com (A.G.); jguineaortega@yahoo.es (J.G.); anafcruz999@gmail.com (A.F.-C.); roberto.alonso@salud.madrid.org (R.A.); emilio.bouza@gmail.com (E.B.); pmunoz@hggm.es (P.M.); 2 Instituto de Investigación Sanitaria Gregorio Marañón, 28007 Madrid, Spain; echamorro@salud.madrid.org (E.C.d.V.); crodriguezg.hgugm@salud.madrid.org (C.G.R.-G.); 3 Fundación Mutua Madrileña Research Fellowship, 28046 Madrid, Spain; 4 Pharmacy Department, Hospital General Universitario Gregorio Marañón, 28007 Madrid, Spain; 5 CIBER Enfermedades Respiratorias, CIBERES (CB06/06/0058), 28029 Madrid, Spain; 6 Departamento de Medicina, Facultad de Medicina, Universidad Complutense de Madrid, 28040 Madrid, Spain

**Keywords:** antifungal stewardship, antifungals, biomarkers, invasive fungal infections, solid organ transplant, oncologic patients, 1,3 β-d-glucan

## Abstract

The implementation of 1,3 β-d-glucan (BDG) has been proposed as a diagnostic tool in antifungal stewardship programs (ASPs). We aimed to analyze the influence of serum BDG in an ASP for oncologic patients and solid organ transplant (SOT) recipients. We conducted a pre–post study. In the initial period (PRE), the ASP was based on bedside advice, and this was complemented with BDG in the post-period (POST). Performance parameters of the BDG assay were determined. Antifungal (AF) use adequacy was evaluated using a point score. Clinical outcomes and AF costs were also compared before and after the intervention. Overall, 85 patients were included in the PRE-period and 112 in the POST-period. Probable or proven fungal infections were similar in both groups (54.1% vs. 57.1%; *p* = 0.67). The determination of BDG contributed to improved management in 75 of 112 patients (66.9%). The AF adequacy score improved in the POST-period (mean 7.75 vs. 9.29; *p* < 0.001). Median days of empiric AF treatment was reduced in the POST-period (9 vs. 5 days, *p* = 0.04). All-cause mortality (44.7% vs. 34.8%; *p* = 0.16) was similar in both periods. The cost of AF treatments was reduced in the POST-period with a difference of 779.6 €/patient. Our data suggest that the use of BDG was a cost-effective strategy that contributed to safely improving the results of an ASP for SOT and oncologic patients.

## 1. Introduction

The use of antifungal (AF) agents is currently increasing [1,2], especially in certain high-risk populations, such as critically ill patients, solid organ transplant (SOT) recipients, and patients with solid tumors or hematologic malignancies. Antifungal stewardship programs (ASPs) have been proposed as an opportunity to optimize antifungal use [3,4,5,6,7]. Most of the ASPs are exclusively based on restrictive prescription strategies or pharmacy alerts, and few of them are based on bedside interventions [8,9,10,11,12]. While different models of ASPs have been designed for intensive care units (ICUs) and onco-hematology units [13,14], not much attention has been paid to SOT and oncologic units.

Implementing ASPs in this population is challenging, due to their high invasive fungal infection (IFI) incidence; the risk of drug-to-drug interactions, especially considering that they receive immunosuppressive drugs; and the risk of organ toxicity. Moreover, previous studies have demonstrated that even when these patients are treated by highly specialized physicians, there is poor compliance with IFI diagnostic and therapeutic guidelines [15,16].

Infection biomarkers have already demonstrated that they are helpful in the treatment of septic patients, and that they have a role in antibiotic stewardship programs [17,18,19]. Fungal biomarkers have emerged as new diagnostic tools that could also be helpful in ASPs. In recent years, our group [20] and other groups [21,22,23] have been evaluating the impact of introducing these fungal biomarkers, such as (1,3) β-d-glucan (BDG) and a *Candida albicans* germ-tube antibody IFA IgG assay (CAGTA), as part of an ASP [24] in ICUs and other populations, different from those used with oncologic and SOT patients.

The main objective of this study was to evaluate the impact of introducing BDG to an already running bedside ASP [12], focusing on oncologic patients and SOT recipients with empiric or targeted AF treatment. We evaluated the impact of the introduction of the BDG in terms of AF use adequacy, clinical outcomes, and AF costs. In addition, we evaluated the performance (sensitivity, specificity, and positive and negative predictive value) of BDG in both populations.

## 2. Materials and Methods

### 2.1. Study Setting and Patient Population

This study was conducted in a 1250-bed tertiary care hospital in Madrid, Spain. Our institution is a referral center for solid organ transplantation (liver, heart, and kidney) and for oncologic patients (immunotherapy, clinical trials). Overall, 135 patients received a SOT per year in our institution (65 renal transplants, 50 liver transplants, and 20 heart transplants per year). The Oncology Department has extensive experience in chemotherapy treatment of solid tumors and contribution to clinical trials. Overall, 330 clinical trials were started between 2010 and 2018, involving 1840 patients.

An ASP has been running in our hospital since 2010 involving several non-compulsory interventions. These interventions include: educational programs, surveillance of AF use, an electronic prescription system with pharmacy alerts to prescribers, a bedside assessment of all restricted AF prescriptions, and a multidisciplinary group of physicians (including microbiologists, infectious diseases specialists, and clinical pharmacists) working together to improve the diagnosis and management of fungal infections and to ensure the quality of AF prescriptions [6]. Since 2014, we have routinely performed the determination of serum BDG after starting antifungal treatment as a tool to improve the IFI diagnosis and to safely stop empiric antifungal therapy to avoid unnecessary treatments, both in the ICU and non-ICU wards, based on the high negative predictive value of this strategy previously published by our group [24].

### 2.2. Study Design and Data Collection

We performed a pre–post study in all adult (≥18 years old) hospitalized patients with a solid tumor or SOT recipients who received systemic AF therapy either as empirical or targeted treatment. Antifungals available at our institution at the time of our study were: fluconazole, micafungin, caspofungin, anidulafungin, liposomal amphotericin B (L-AmB), voriconazole, and posaconazole.

During the initial period (PRE)—from October 2011 to August 2014—the ASP was based on bedside advice provided by expert infectious disease specialists using a pre-established protocol. In the intervention period (POST)—from September 2014 to July 2017—advice was complemented with the result of serum BDG performed, at least, on days +1, +3, and +5 of AF treatment for each patient. AF use adequacy, AF consumption, and clinical outcomes were compared before and after the intervention.

We prospectively collected the following data: (i) patient characteristics (age; gender; comorbidities; severity of the underlying medical conditions, using the Charlson comorbidity index; presence of IFI risk factors, namely underlying immunosuppression, central venous catheter, surgery in the last 3 months, corticosteroids, total parenteral nutrition (TPN), and continuous renal replacement therapy); (ii) fungal disease (indication of antifungal prescription, clinical and radiological signs, microbiological and histopathological findings, culture and susceptibility test results, and serological test results, i.e., *Aspergillus* galactomannan, BDG); (iii) antifungal therapy (drug prescribed, including the dosage, administration route, and date of initiation and end of therapy]); (iv) adequacy of antifungal use; and (v) antifungal days of therapy (DOTs) and cost.

### 2.3. Definitions

The criteria for invasive candidiasis included candidemia and deep-seated candidiasis, as defined by the European Society of Clinical Microbiology and Infectious Diseases (ESCMID) guideline for the diagnosis and management of candida diseases [25]. Deep-seated candidiasis may stem from hematogenous dissemination or direct introduction of *Candida* to a sterile site. These infections may be localized, spread to contiguous sites, or lead to secondary candidemia. For the purpose of our study, we applied the following classification: (1) Candidemia in the absence of deep-seated candidiasis; (2) candidemia associated with deep-seated candidiasis; (3) deep-seated candidiasis that is not associated with candidemia, as proposed by Clancy et al. [26].

Multifocal candida colonization was defined as the isolation of *Candida* spp. from at least two samples (surveillance and clinical samples).

The criteria for invasive filamentous fungal infection diagnosis were based on the 2008 version of the European Organization for Research and Treatment of Cancer/Mycoses Study Group (EORTC/MSG) [27]. When classifying invasive aspergillosis cases, a positive *Aspergillus* spp. PCR in bronchoalveolar lavage (BAL) was also considered as a type C microbiologic criterion.

AF therapy was categorized as follows: empirical treatment for a suspected infection, and targeted treatment for a documented fungal infection.

IFI-related death was defined as death with current signs of IFI [28].

The criteria used to define the appropriateness of AF prescription were adopted from the treatment guidelines of the Infectious Diseases Society of America and the European Society of Clinical Microbiology and Infectious Diseases [25,29] and according to our local guidelines that take into account our susceptibility testing.

The adequacy of AF use was evaluated using a point score previously defined and published [6]. This score provides a maximum of 10 points (Table 1) and assigns a relative weight to each of the six evaluated items (indication, selection, dosage, microbiological adjustment, sequential treatment, and duration) that were judged at the end of AF therapy. We decided to assign more impact (0 or 2 points) to mistakes that could imply a major risk for the patient (prescription of an unneeded antifungal agent) or to aspects that were clear intervention targets (lack of adjustment following receipt of microbiological information or excessive duration of treatment). Less detrimental mistakes, such as incorrect dosage or lack of switching to an oral form, were given a smaller impact (0 or 1 point) in the global score. In the case of drug selection, we decided to offer three possible values: prescription of a drug that did not cover the suspected fungal pathogen (major mistake: 0 points); prescription of a drug that covered the pathogen, although this was not optimal according to our local guidelines (minor mistake: 1 point), and; perfect selection of the antifungal drug (2 points).

For adequate dosage adjustments, hepatic and/or renal dysfunction, weight, and drug interactions or voriconazole serum levels (when available) were also considered. When adjusting for renal dysfunction, we estimated the glomerular filtration rate (GFR) according to the Modification of Diet in Renal Disease (MDRD), while for hepatic dysfunction evaluation, we considered the Child–Pugh score.

Any prescription with a global AF adequacy score less than 10 points was judged as inappropriate. This score was applied after patient discharge.

The number of DOTs and drug costs (€) were calculated on the basis of the actual dose administered and the purchase price for the institution after markup by the pharmacy, excluding administration costs. All data were collected by two investigators and recorded using a data collection tool.

### 2.4. Laboratory Procedures

Serological detection of BDG was performed using the Fungitell^®^ assay kit (Associates of Cape Cod) according to the manufacturer’s instructions. Results were analyzed with a BioTek ELX808TM Microplate Reader and GEN5 Software (BioTek U.S., Winooski, VT, USA) and considered positive when the values were ≥80 pg/mL [24]. GM testing was performed using Platelia™ *Aspergillus* (Bio-Rad Laboratories, Hercules, CA, USA) with a cut-off value of ≥0.5 in serum and ≥1.0 in BAL. Both serum GM and BDG and BAL GM (if possible) were performed in cases of suspected *Aspergillus* infection.

Culture of respiratory samples was performed upon clinical request for mould infection diagnosis on Sabouraud dextrose agar and Brain Heart Infusion (BHI) agar.

Blood cultures (BC) for candidemia diagnosis were obtained through venepuncture using standard procedures and processed in clinical laboratories with the BD Bactec FX (Becton, Dickinson, Sparks, MD, USA).

The diagnosis of deep-seated candidiasis was made by the isolation of *Candida* spp. from normal sterile body fluids or peritoneal fluid obtained during surgery, or by percutaneous aspiration, or by drainage inserted for <24 h. Colonization was defined as the recovery of *Candida* spp. from non-sterile sites (urine, stool, drainage >24 h), independently of the presence of signs or symptoms attributable to the clinical suspicion of invasive candidiasis.

For *Pneumocystis jirovecii* diagnosis, respiratory samples were performed by indirect immunofluorescent antibody (IIFA) assay able to detect cysts and trophic forms (MONOFLUO *P. jirovecii* IFA BIO-RAD, Marnes-la-Coquette, France) according to the manufacturer’s instructions. The results were reviewed and validated for an expert microbiologist. Since 2015, molecular diagnosis has been implemented and all samples were performed by a qualitative real-time PCR, RealCycler PJIR kit^®^ (Progenie Molecular, Valencia, Spain). RT-PCR targets the *P. jirovecii* mitochondrial large subunit ribosomal RNA (mtLSUrRNA) coding region and uses a hydrolysis probe with a FAM fluorophore. DNA was extracted from respiratory samples using a QIAamp DNA minikit (Qiagen, Hilden, Germany) according to the manufacturer’s instructions in a MagnaPure Compact automated system (Roche^®^).

### 2.5. Statistical Analysis

Data were entered into a database using Microsoft Access^®^. The qualitative variables appear with their frequency distribution. Normally distributed quantitative variables are expressed as the mean and standard deviation (SD); non-normally distributed variables are expressed as the median and interquartile range (IQR). Groups were compared using the χ^2^ test for categorical variables and the Mann–Whitney U-test for continuous variables. The sensitivity, specificity, positive predictive value (PPV), and negative predictive value (NPV) of the BDG were calculated for each population of oncologic and SOT patients. Validity values were calculated with a 95% CI. A *p* value of <0.05 was considered significant.

IBM SPSS^®^ software package (SPSS Inc. Version 20.0, Chicago, IL, USA) was used for the statistical analysis.

## 3. Results

### 3.1. Demographic and Clinical Characteristics of Patients

Overall, 197 patients were included—85 patients in the PRE-period, and 112 in the POST-period. The main differences in demographic and clinical characteristics between both periods are described in Table 2. Both groups differed in the rate of solid organ transplant recipients (40% vs. 24.1%; *p* = 0.02) and oncological patients (60% vs. 75.9%; *p* = 0.02), as shown in Table 2. Concerning the comorbidity observed in both periods, in the PRE-period there were more patients with renal insufficiency (30.6% vs. 17.9%; *p* = 0.04), liver dysfunction (30.6% vs. 13.4%; *p* = 0.003), and secondary hematologic disease (10.6% vs. 2.7; *p* = 0.02). Classical risk factors were similar in both periods, except for a higher percentage of neutropenia in the PRE period (14.1% vs. 5.4; *p* = 0.03) and a higher percentage of TPN (28.0% vs. 50%; *p* = 0.01) in the POST-period, probably due to a greater proportion of patients with an abdominal surgery (31.8% vs. 46.4%; *p* = 0.04) and a longer intensive care unit stay (more than 7 days) (20% vs. 34.8%; *p* = 0.02).

Overall, probable or proven fungal infections were similar in both periods (54.1% vs. 57.1%; *p* = 0.67). Distribution between proven and probable infection was similar during both the PRE and POST periods: 60% vs. 70.3% (*p* = 0.26), and 33.3% vs. 29.7% (*p* = 0.68), respectively.

The distribution of candidemia, invasive candidiasis, aspergillosis, mucormycosis, *Pneumocystis jirovecii* pneumonia (PJP), and scedosporiasis was also similar in both periods, as described in Table 3. We isolated a total of 271 fungal species from clinical samples corresponding to 142 patients, some of them without IFI criteria diagnosis, where it was considered that colonization occurred. According to EORTC/MSG 2008 (the diagnostic standard at the time of the study), one *Pneumocystis jirovecii* pneumonia was diagnosed based on a typical clinical and radiological presentation, and only one positive BDG of ≥80 pg/ml as unique microbiological criteria.

No differences were found in the distribution of the fungal species.

Regarding antifungal therapy indication, empirical treatments correspond to 42.4% in the PRE-period and 65.2% in the POST-period (*p* = 0.001), and targeted treatments correspond to 57.6% in the PRE-period and 34.8% in the POST-period (*p* = 0.001).

There was a decrease in the use of caspofungin in the POST-period (21.2% vs. 6.2%; *p* = 0.002), while fluconazole prescriptions increased in the POST-period (18.8% vs. 45.5%; *p* < 0.001). The use of other antifungal agents was similar in both periods.

Median days of treatment for empirical antifungal courses decreased from 9 (IQR 4-14) in the PRE-period to 5 (IQR 2-11) in the POST-period (*p* = 0.04).

Overall, 19 patients received previous antifungal prophylaxis before the clinical event that triggered the empiric or targeted antifungal prescription. In the PRE-period, six out of eight patients that received prophylaxis finally developed an IFI (66.7%); three of them were SOT recipients and one was an oncologic patient. A similar proportion (54.5%) of patients under prophylaxis developed an IFI in the POST-period (6 out of 11), corresponding to one SOT recipient and five oncologic patients. The main antifungal agents used for prophylaxis were fluconazole (41.2%) and micafungin (35.2%).

Regarding the outcome, all-cause mortality was similar in both periods (44.7% vs. 34.8%; *p* = 0.16), and no observable differences were found for IFI-related mortality (10.6% vs. 4.5%; *p* = 0.17).

### 3.2. Differences in Clinical Characteristics between Oncology and Transplant Recipients

We have compared the differences between oncologic patients and SOT recipient (Table S1). Oncologic patients were older than SOT recipients (63.4 vs. 54.2 years old; *p* < 0.001). However, SOT recipients had more comorbidities, especially renal failure (12.5% vs. 47.5%; *p* < 0.001), diabetes mellitus (16.2% vs. 41%; *p* < 0.001), and hepatic insufficiency (5.1% vs. 55.7%; *p* < 0.001).

Regarding IFI risk factors, cancer patients had received more TPN (49.3% vs. 26.2%; *p* = 0.002) and neutropenia (13.2% vs. 0%; *p* = 0.003). On the other hand, SOT patients who had a greater rate of long ICU stays >7 days (23.5% vs. 39.3%; *p* = 0.02) had received more broad-spectrum antibiotics (54.4% vs. 72.8%; *p* = 0.01), required more hemodialysis (54.4% vs. 72.8%; *p* = 0.01), and extracorporeal membrane oxygenation (ECMO) therapy (0% vs. 12.2%; *p* < 0.001). No differences were found in the fungal infection incidence, the use of empirical or targeted treatment, or its duration. As for the type of antifungals used, in the group of cancer patients, there was more use of fluconazole (44.1% vs. 11.5%; *p* < 0.001) and candins in the SOT group (39.7% vs. 62.3%; *p* = 0.003).

As expected, SOT recipients had received more AF prophylaxis (6.6% vs. 16.4%); *p* = 0.03). There were no differences with respect to all-cause mortality and IFI-related mortality.

### 3.3. Diagnostic Performance of Serum BDG

Overall, 299 BDG tests were performed in the POST-period (180 positive results and 119 negative results). The determination of BDG contributed to improving management in 75 of 112 patients (66.9%), by means of helping to stop the AF treatments in 35 patients (46.7%) without IFI and supporting the diagnosis in 35 patients (46.7%). It also contributed to change to another AF treatment in four patients (5.3%) and association with a second AF agent in one patient (1.3%). The sensitivity, specificity, and predictive values of BDG are shown in Table 4. We diagnosed two cases of IFI with BDG: a case of intra-abdominal candidiasis (in which there was no possibility of obtaining an intra-abdominal clinical sample for culture and had sterile blood cultures), and a *Pneumocystis jirovecii* pneumonia case (in which direct immunofluorescence was negative in sputum and no PCR was performed).

We studied the differences between both populations (oncologic and SOT) and observed some variations. In oncologic patients, the sensitivity was lower than in SOT recipients (77.4% vs. 92.6%), and the negative predictive value (NPV) was also lower (65.8% vs. 88.9%).

We also estimate the diagnostic performance of BDG in neutropenic patients (n = 6), observing that they presented high sensitivity and specificity, as well as NPV and PPV.

We analyzed all patients who had empirical treatment, in whom all three determinations of BDG were negative, and who finally had a fungal infection. A total of 7 out of 40 patients (17.5%) eventually had an IFI: six had invasive candidiasis, and one had a mold infection.

### 3.4. Adequacy of Antifungal Therapy

The AF adequacy score improved in the POST-period (mean 7.75 vs. 9.29; *p* < 0.001), mainly due to higher adequacy of antifungal indication (88.2% vs. 98.2%; *p* = 0.004), better microbiological adjustment (69.4% vs. 96.4%; *p* < 0.001), a higher percentage of patients that switched to oral when it was possible (85.9% vs. 98.2%; *p* = 0.001), and a higher percentage of optimal treatment duration (57.6% vs. 83.9%; *p* < 0.001). These results are described in Table 5.

### 3.5. Antifungal Consumption and Potential Saved Costs

The median number of DOTs used per patient was 16.0 (9.0–26.0) in the PRE-period and 16.0 (8.3–24.8) in the POST-period. The direct acquisition cost of AF was 2777.3 (1388.7–4876.2) and 1997.7 (54,9–4968.5) €/patient, respectively. The cost of AF treatments was reduced in the POST-period, with a difference of 779.6 €/patient.

As previously shown in Table 2, the duration of empirical AF treatments was significantly reduced in the POST-period, from 9 (4–14) to 5 (2–11) DOTs (*p* = 0.04), whereas no difference was observed for targeted therapy.

## 4. Discussion

Our study is the first to evaluate the potential role of serum BDG as a diagnostic tool used within a bedside ASP for solid organ transplant recipients and oncologic patients. Previous experience with ASPs shows us that the most difficult task is to safely stop antifungal agents that were prescribed in empirical cases. Reducing the use of unnecessary prescribed AF treatments may decrease the complications associated with toxicity, drug-to-drug interactions, the rise of resistant infections, and costs (direct and indirect costs). Non-culture-based diagnostic techniques have shown their benefit in ASPs, but the peculiarities of each population must be taken into account for their correct application.

Non-compulsory ASPs have already demonstrated that they are cost-effective and could improve the quality of antifungal prescriptions without having a deleterious impact on patient outcomes [30]. Performing ASPs in immunosuppressed patients is challenging due to their high risk of IFIs and associated morbidity and mortality.

In our study, we observed that the proportion of SOT recipients is significantly lower in the most recent period (POST-period). This may be due to the improvement in AF prophylaxis strategies that had already reduce the incidence of IFIs, at least at our center.

During the POST period, we saw more patients requiring abdominal surgery, longer ICU stay (more than 7 days) and total parenteral nutrition, so we believe that was the reason for a higher rate of empirical treatments. We also considered that educational activities, local guidelines, implementation of new diagnostic methods (including the BDG), and expert clinical advice have changed the way our clinicians prescribe antifungals. The awareness of the importance of starting early AF treatment by our hospital prescribers, could also explain the increase in empirical treatments. The role of BDG in addressing the problem of excessively long empirical treatments has already been explored in other previous studies considering other populations (ICU patients, haemato-oncologic patients) [21,31,32]. We had previously observed that a combination of BDG and CAGTA performed on days 0, 3, and 5 during empirical antifungal therapy had a very high negative predictive value (97% for the entire population and 100% in ICU patients) [24]. In our study, we found that when a similar strategy was applied to SOT recipients, where the rate of documented fungal infection was similar (48.1%), the sensitivity of serum BDG was 93.5%, and the NPV was 92.9%. With these results, we can advise that AF treatments be stopped with a certain degree of safety in cases with negative BDG in several determinations.

On the other hand, our oncological patients had a higher rate of documented IFI (60%), with more risk factors for this type of infection (permanent central venous catheter for a chemotherapy and requirement of TPN). Therefore, the NPV for this population was only 65.8%. Conversely, the PPV was greater (87.3%), and BDG would have real value in being able to confirm the disease when the result is positive, but not in stopping empirical treatment when the BDG results are negative.

In addition, we have observed an increase in the use of fluconazole, which translates to the positive influence of our previous ASP interventions that already convinced prescribing physicians of the importance and benefit of early de-escalation to lower spectrum antifungals. It is important to highlight that in our setting, the fluconazole resistance rate in invasive candidiasis is still less than 5% [33].

The application of BDG to optimize antifungal therapy as part of our bedside intervention did not affect patient prognosis. IFI-related mortality and all-cause mortality did not increase in the POST-period. Overall, the adequacy score of antifungal prescriptions was found to improve after BDG implementation.

Finally, this strategy is also cost-effective, allowing us to save 779.6 € per patient. After analyzing the BDG performance in both populations, we concluded that it could be helpful to reduce the length of unnecessary antifungal empiric therapy in solid organ transplant recipients. However, it is not that valuable in oncology patients due to its low NPV.

Our study is subject to a series of limitations. First, its design as a pre–post study leads to some differences in the clinical characteristics of the patients between both cohorts. Second, the diagnostic sensitivity and specificity of the BDG was evaluated according to a gold standard (EORTC/MSG guidelines) that classifies invasive fungal infections based on a combination of clinical and host factors, and microbiological criteria (which also add up the BDG) which represents an incorporation bias. Third, as this study was performed at a single tertiary care center, the results may not be applicable to other less specialized institutions. Finally, price may differ from the officially established price, owing to discounts negotiated with drug suppliers.

In conclusion, the determination of BDG in SOT recipients and oncological patients is a tool that can be used to stop empirical prescribed AF treatments or to confirm the diagnosis of IFI. This is a cost-effective strategy that safely improves the results of an ASP based on bedside advice in a tertiary care hospital.

## Figures and Tables

**Table 1:** Score for evaluating appropriateness of antifungal (AF) therapy.

Feature	Question	Answer	Points
Indication	Did the patient need an antifungal?	Yes	2
		No	0
Selection	Did the antifungal cover the suspected fungi and was it the first option recommended by the guidelines?	It covered the suspected fungi and was the first option	2
		It covered the suspected fungi but was the alternative option	1
		It did not cover the suspected fungi	0
Dosage ^1^	Was the dosage correct according to the body weight, hepatic and renal function, and potential interactions with other drugs?	Yes	1
		No	0
Microbiological adjustment	Was the antifungal adjusted after microbiological results (identification of microorganism, antifungal susceptibility tests, and indirect tests) became available?	Yes	2
		No	0
Administration route	Was the intravenous route switched to the oral route when possible?	Yes	1
		No	0
Duration	Was the duration of therapy correct according to the guidelines? ^2^	Yes	2
		No	0
Total score (From 0 to 10)			

^1^ Both low and high doses were considered incorrect. Adjustment for renal and hepatic failure and drug-to-drug interactions were also addressed. At the time of the study, monitoring of serum voriconazole and posaconazole was not available. ^2^ Durations that were too short and too long were considered incorrect.

**Table 2:** Demographic and clinical characteristics of 197 included patients.

	PRE-Period	POST-Period	*p*
	n = 85	n = 112	
**Age (years), mean (SD)**	57.1 (±13.7)	63.3 (±13.8)	**0.002**
**Male (%)**	54 (63.5)	78 (69.6)	0.37
**Oncological (%)**	51 (60.0)	85 (75.9)	**0.02**
**Solid Organ Transplant (%) ^1^**	34 (40.0)	27 (24.1)	**0.02**
Liver	21 (61.8)	12 (44.4)	0.18
Cardiac	4 (11.8)	12 (44.4)	**0.004**
Kidney	9 (26.5)	3 (11.1)	0.13
**Comorbidity (%)**			
Heart chronic disease	21 (24.7)	38 (33.9)	0.16
Renal failure	26 (30.6)	20 (17.9)	**0.04**
Respiratory disease	23 (27.1)	25 (22.3)	0.44
DM	19 (22.4)	28 (25.0)	0.67
Neurological disease	9 (10.6)	16 (14.3)	0.44
Liver dysfunction	26 (30.6)	15 (13.4)	**0.003**
Hematologic disease	9 (10.6)	3 (2.7)	**0.02**
HIV	3 (3.5)	1 (0.9)	0.19
Autoimmune disease	1 (1.2)	3 (2.7)	0.46
**Risk factors of IFI**			
TPN	27 (31.8)	56 (50.0)	**0.01**
CVC	52 (61.2)	73 (65.2)	0.56
Urinary catheter	41 (48.2)	47 (42.0)	0.38
Abdominal surgery (last 3 months)	27 (31.8)	52 (46.4)	**0.04**
ICU stay >7 days	17 (20.0)	39 (34.8)	**0.02**
Mechanical ventilation	13 (15.3)	29 (25.9)	0.07
Corticosteroids	30 (35.3)	37 (33.0)	0.74
Pancreatitis	5 (5.9)	9 (8.0)	0.56
Hemodialysis	16 (18.8)	14 (12.5)	0.22
Neutropenia	12 (14.1)	6 (5.4)	**0.03**
Broad spectrum antibiotics	53 (62.4)	66 (58.9)	0.63
Multifocal colonization	14 (16.5)	14 (12.5)	0.43
ECMO therapy	1 (2.9)	4 (3.6)	0.84
**Indication of antifungal therapy**			
Empirical	36 (42.4)	73 (65.2)	**0.001**
Targeted	49 (57.6)	39 (34.8)	**0.001**
**First antifungal treatment**			
Voriconazole	15 (17.6)	12 (10.7)	0.16
Fluconazole	16 (18.8)	51 (45.5)	**<0.001**
Posaconazole	0	1 (0.9)	0.38
Caspofungin	18 (21.2)	7 (6.2)	**0.002**
Anidulafungin	17 (20.0)	26 (23.2)	0.59
Micafungin	12 (14.1)	11 (9.8)	0.35
L-AMB	7 (8.2)	4 (3.6)	0.16
**Duration of first AF treatment—days, median (IQR)**			
Empirical	9 (4–14)	5 (2–11)	**0.04**
Targeted	8 (3–17.5)	9 (4–19)	0.60
**Previous antifungal prophylaxis**	8 (9.4)	11 (9.8)	0.92
**All-cause mortality**	38 (44.7)	39 (34.8)	0.16
**IFI-related mortality**	9 (10.6)	5 (4.5)	0.17

Legend: AF, antifungal; CVC, central venous catheter; DM, diabetes mellitus; ECMO, extracorporeal membrane oxygenation; HIV, human immunodeficiency virus; ICU, intensive care unit; IFI, invasive fungal infection; IQR, interquartile range; L-AMB, liposomal-amphotericin B; SD, standard deviation; TPN, total parenteral nutrition. ^1^ Seven patients with hepatocellular carcinoma were also patients who received a liver transplant, so they were considered in the solid organ transplant group. *p* values marked in bold indicate numbers that were statistically significant (*p* < 0.05).

**Table 3:** Description of invasive fungal infections.

	PRE-Period	POST-Period	*p*
	n = 85	n = 112	
**Invasive fungal infection diagnosis**	46 (54.1)	64 (57.1)	0.67
Candidemia in the absence of deep-seated candidiasis ^1^	13 (15.3)	23 (20.5)	0.35
Candidemia and deep-seated candidiasis	5 (5.9)	6 (5.4)	0.87
Deep-seated candidiasis	13 (15.3)	20 (17.9)	0.63
Invasive aspergillosis	11 (12.9)	10 (8.9)	0.37
Mucormycosis	2 (2.4)	1 (0.9)	0.41
*Pneumocystis jirovecii* pneumonia	2 (2.4)	3 (2.7)	0.88
Disseminated scedosporiasis	0	1 (0.9)	0.38
**Species (218 isolates)**			
*Candida albicans*	27 (31.8)	43 (38.4)	0.34
Non-*albicans Candida*	32 (37.6)	31 (27.7)	0.13
*Aspergillus* spp.	12 (14.1)	15 (13.4)	0.88
Mucorales	2 (2.4)	1 (0.9)	0.41
*Pneumocystis jirovecii*	2 (2.4)	2 (1.8)	0.78
*Lomentospora prolificans*	0	1 (0.9)	0.38

^1^ 13/13 were catheter-related candidemia in the PRE period and 20/23 in the POST period, *p* = 0.63.

**Table 4:** Diagnostic performance of serum BDG.

	Documented Fungal Infections (n, %)	S (%) 95% CI	Sp (%) 95% CI	PPV (%) 95% CI	NPV (%) 95% CI
Oncologic (n = 85)	51 (60.0)	77.4 (68.7–84.7)	79.4 (67.3–88.5)	87.3 (80.7–91.8)	65.8 (57.3–73.4)
SOT (n = 27)	13 (48.1)	92.6 (75.7–99.1)	55.2 (35.7–73.5)	65.8 (55.9–74.5)	88.9 (66.9–96.9)
Empirical treatment (n = 73)	30 (41.1)	79.1 (67.4–88.1)	69.4 (58.4–78.9)	67.1 (59.1–74.2)	80.8 (72.1–87.3)
Neutropenic patients (n = 6)	4 (66.7)	87.5 (47.3–99.7)	93.3 (68.1–99.8)	87.5 (50.8–97.9)	93.3 (69.0–98.9)

Legend: BDG, (1,3) β-d-glucan; CI, confidence interval; IC, invasive candidiasis; NPV, negative predictive value; S, sensitivity; Sp, specificity; SOT, solid organ transplant; PPV, positive predictive value.

**Table 5:** Evaluation of antifungal adequacy.

Adequacy Features	PRE-Period	POST-Period	*p*
	n = 85 (%)	n = 112 (%)	
Adequate indication	75 (88.2)	110 (98.2)	**0.004**
Adequate selection	79 (92.9)	109 (97.3)	0.14
Adequate dosage	68 (80)	99 (88.4)	0.10
Adequate microbiological adjustment	59 (69.4)	108 (96.4)	**<0.001**
Adequate administration route	73 (85.9)	110 (98.2)	**0.001**
Adequate duration of therapy	49 (57.6)	94 (83.9)	**<0.001**
Total adequacy score, mean (SD)	7.75 (±2.1)	9.29 (±1.27)	**<0.001**

SD, Standard Deviation. *p* values marked in bold indicate numbers that were statistically significant (*p* < 0.05).

## Data Availability

The data presented in this study are available on request from the corresponding author. The data are not publicly available due to privacy.

## Supplementary Materials

The following are available online at https://www.mdpi.com/2309-608X/7/1/59/s1, Table S1: Differential demographic and clinical characteristics between oncological and solid organ transplant recipients.
