# Supplementary material for: Utility of 1,3 β-d-Glucan Assay for Guidance in Antifungal Stewardship Programs for Oncologic Patients and Solid Organ Transplant Recipients
Source: J Fungi (Basel). 2021 Jan 17;7(1):59. doi: 10.3390/jof7010059 (PMC7830184; doi:10.3390/jof7010059)
Supplement: Supplementary file 1 [file jof-07-00059-s001.pdf]

Table S1. Differential demographic and clinical characteristics between oncological and solid organ transplant recipients.

	ONCO<br>n = 136	SOT<br>n = 61	*p*
**Age (years), mean (SD)**	63.4 (±14.5)	54.2 (±10.5)	**<0.001**
**Male (%)**	86 (63.2)	46 (75.4)	0.09
**Comorbidity (%)**			
Heart chronic disease	36 (26.5)	23 (37.7)	0.11
Renal failure	17 (12.5)	29 (47.5)	**<0.001**
Respiratory disease	36 (26.5)	12 (19.7)	0.30
DM	22 (16.2)	25 (41)	**<0.001**
Neurological disease	20 (14.7)	5 (8.2)	0.20
Liver dysfunction	7 (5.1)	34 (55.7)	**<0.001**
Hematologic disease	7 (5.1)	5 (8.2)	0.41
HIV	3 (2.2)	1 (1.6)	0.79
Autoimmune disease	4 (2.9)	0	0.18
**Risk factors of IFI**			
TPN	67 (49.3)	16 (26.2)	**0.002**
CVC	87 (64.0)	38 (62.3)	0.82
Urinary catheter	53 (39.0)	35 (57.4)	**0.02**
Abdominal surgery (last 3 months)	54 (39.7)	25 (41.0)	0.86
ICU stay >7 days	32 (23.5)	24 (39.3)	**0.02**
Mechanical ventilation	25 (18.4)	17 (27.9)	0.13
Corticosteroids	42 (30.9)	25 (41)	0.17
Pancreatitis	8 (5.9)	6 (9.8)	0.32
Hemodialysis	9 (6.6)	21 (34.4)	**<0.001**
Neutropenia	18 (13.2)	0	**0.003**
Broad spectrum antibiotics	74 (54.4)	45 (72.8)	**0.01**
Multifocal colonization	17 (12.5)	11 (18.0)	0.30
ECMO therapy	0	5 (12.2)	**<0.001**
**Indication of antifungal therapy**			
Empirical	74 (54.4)	35 (57.4)	0.70
Targeted	62 (45.6)	26 (42.6)	0.70
**Invasive fungal infection**	78 (57.4)	31 (50.8)	0.39
Invasive candidiasis	20 (14.7)	13 (21.3)	0.25
Invasive mold disease	16 (11.8)	13 (21.3)	0.08
**First antifungal treatment**			
Voriconazole	18 (13.2)	9 (14.8)	0.77
Fluconazole	60 (44.1)	7 (11.5)	**<0.001**
Candins	54 (39.7)	38 (62.3)	**0.003**
L-AMB	4 (2.9)	7 (11.5)	**0.02**
**Duration of first AF treatment—days, median (IQR)**			
Empirical	7 (3.0-12.7)	5 (2.0-10.0)	0.28
Targeted	6 (2.0-18.0)	10 (2.5-21.5)	0.39
**Previous antifungal prophylaxis**	9 (6.6)	10 (16.4)	**0.03**
**All-cause mortality**	58 (42.6)	19 (31.1)	0.13
**IFI-related mortality**	48/58 (82.8)	15/19 (78.9)	0.71

Legend: AF, antifungal; CVC, central venous catheter; DM, diabetes mellitus; ECMO, extracorporeal membrane oxygenation; HIV, human immunodeficiency virus; ICU, intensive care unit; IFI, invasive fungal infection; IQR, interquartile range; L-AMB, liposomal-amphotericin B; ONCO, oncological patients; SD, standard deviation; SOT, solid organ transplant recipients; TPN, total parenteral nutrition. *p* values marked in bold indicate numbers that are statistically significant ($p < 0.05$).
